# Developing an Integrated Evaluation Model for Physician Comprehensive Workload Tethered to Outpatient Practice: An Empirical Study From China

**DOI:** 10.3389/fpubh.2022.847613

**Published:** 2022-05-19

**Authors:** Dehe Li, Yinhuan Hu, Sha Liu, Chuntao Lu, Yeyan Zhang, Jinghan Zhou, Jiayi Li, Zemiao Zhang

**Affiliations:** ^1^School of Medicine and Health Management, Tongji Medical College, Huazhong University of Science and Technology, Wuhan, China; ^2^Jingmen No. 2 People's Hospital, Jingmen, China

**Keywords:** physician comprehensive workload, outpatient practice, evaluation framework, integrated model, China

## Abstract

**Background:**

Previous studies, often simply using either objective workload or mental workload as a measure of physician workload in various healthcare settings might have failed to comprehensively reflect the real workload among physicians. Despite this, there is little research that further explores a comprehensive workload evaluation framework with the integration of objective workload and mental workload to describe their comprehensive workload.

**Methods:**

A comprehensive evaluation framework for physician workload was proposed based on the combination of objective workload and task-level mental workload also with the consideration of quality of provided medical services and served patient complexity; and accordingly, an integrated evaluation model for physician comprehensive workload (PCW) tethered to outpatient practice was developed and further applied to perform a PCW analysis using cross-sectional data on outpatient workload of 1,934 physicians mainly from 24 hospitals in 6 provinces in Eastern, Central, and Western China. Multiple linear regression and multinomial logistic regression analyses were established to identify significant factors influencing the PCW.

**Results:**

Overall, the average score of PCW tethered to outpatient practice Chinese physicians experienced was 811.30 (SD=494.98) with concentrating on between 200 and 1,200. Physicians who were female, from Eastern or Western China, and those who worked >60 h per week and longer outpatient hours per week were more likely to experience a higher PCW. 11.2% of participating physicians were identified as very high PCW physicians, compared with 11.6% as low PCW physicians, 45.5% as medium PCW physicians and 30.7% as high PCW physicians. Those who were female, older, from Western China, those who had lower educational levels, lower professional titles and longer working years in the current institution, and those who worked in tertiary A hospitals and Internal or Surgical, and worked >60 h per week and longer outpatient hours per week were more likely to be very high PCW physicians.

**Conclusions:**

Our work has a potential application for comprehensively assessing physician workload tethered to outpatient practice and could provide a solid foundation for hospital managers to further accurately determine and identify physicians with high workload, who would otherwise be missed in either objective workload or mental workload.

## Introduction

Currently, heavy physician workload is a common challenge internationally. With a growing aging population with chronic and age-related diseases, along with subsequently ever increasing health care demands worldwide (1), especially in China (2), alarming increasing trends in physician workload have attracted much attention from health care providers, researchers and decision makers in recent years, owing to lack of a proportional growth in the number of physicians (3–5). Increased physician workload is associated with their worse occupational health (6, 7), inferior quality of medical care and even patient harm (5, 7–9). Healthcare organizations may also experience adverse effects such as low productivity, increased cost (10), and increased use of sick leave and turnover rates among physicians (9). Hence, there is an increasingly urgent need for assessing and managing physician workload to mitigate the potential negative effects caused by increased physician workload.

In China, overwhelming workload in physicians has become one of the major concerns for current health care system (11), which has become one of the main sources of their high work pressure; and the prominent physician shortage issue in China [2.04 practicing physicians per 1,000 people in 2017 (12), compared to the international average of 3.5 (13)] along with extremely unbalanced distribution of high-quality physicians between urban and rural areas and between regions may further lead to a much heavier workload for Chinese physicians, especially in tertiary A hospitals in big cities. As the hierarchical diagnosis and treatment system of China has not yet achieved effective triage of patients (4), also with ever-increasing patient demands for high-quality medical services, physicians especially in large general hospitals tend to have an increasingly heavy outpatient workload, with a greater number of outpatients seen daily (4) (even up to 100 patients a day), resulting in a more limited time available for interaction with their patients on average and patient dissatisfaction with the medical services they provided. Not surprisingly, data from a recent national survey from 136 public tertiary hospitals across 31 provinces in China showed that most physicians worked >10 h per day (an average of 10 h) to manage outpatients and inpatients, and almost two-thirds reported a heavy workload (14); and the latest data from Chinese Physician Practice Situation White Paper in 2018 also reported 85.41% of Chinese physicians worked > 40 h per week, and even 32.69% worked > 60 h per week (4). High levels of workload have also been recognized as a contributor to high error rates, and even patient and visitor violence. In addition to this, excess loads of the entire health care delivery system can be passed directly to or through the outpatient clinics, adding to the complexity and strain already being experienced, thereby becoming a location with high risks of suffering from medical disputes, and even violence against healthcare professionals. Under such circumstances, hospitals seek to determine individuals among physicians, who need interventions in priority, so as to expand effective supply of their outpatient services and continuously improve the quality and efficiency of medical services and patient safety while lightening their workload.

Chinese researchers have paid increasing attention to the measurement and evaluation of Chinese physicians' workload in recent years. Currently, the National Health Commission of the People's Republic of China and National Administration of Traditional Chinese Medicine jointly issued a document in 2020, clearly pointing out that it's necessary to scientifically calculate medical workers' workload and further allocate the human resources rationally, so as to avoid long-term overload and reduce their physical and mental pressure (15). Hence, it's of great importance to establish a comprehensive evaluation model for physician workload to further strengthen the assessment and management of Chinese physicians' workload. However, existing research on physician workload in China with slow progress, reflects the work burden of outpatient physicians mainly using several objective workload indicators (for example, the number of outpatients seen, and working hours per week), with less consideration of their subjective psychological experiences (that is, mental workload) (7). Such metrics for evaluating physician workload are inadequate. Physicians are generally simultaneously exposed to both physical and mental workload stress in outpatient practice, necessitating simultaneously monitoring of physical and mental workload for more comprehensive workload evaluation. There is therefore an urgent need for a comprehensive workload evaluation framework to describe their comprehensive workload that integrates both their objective workload and mental workload.

Given that the impending issues that outpatient clinics are facing underscore the need for an approach of comprehensively assessing physician workload, the main objective of this study is to explore to develop an integrated evaluation model for comprehensive assessments of physician workload tethered to outpatient practice. The specific objective is to perform a physician comprehensive workload analysis according to our developed integrated evaluation model for physician workload using cross-sectional data on outpatient physician workload from a nationwide survey. Our current understanding of the integrated evaluation model for comprehensively assessing physician workload is rather limited. Previous research often simply adopted either objective workload or mental workload as the measure of physicians' workload in various healthcare settings. Such kind of study, although important might have failed to comprehensively reflect their real workload. This study fills the literature by developing an integrated evaluation model for physician workload, and can help provide a solid foundation for hospital managers to accurately identify those with high workload as individuals who need interventions in priority, who would otherwise be missed in either objective workload or mental workload.

### Literature Review

#### Concepts and Definitions of Workload

Workload is a multidimensional and multifaceted concept that remains inconsistent definitions worldwide (7), literally meaning the amount of workloads a human operator undertakes per unit time. Even without consensus on a definition, workload has become a topic of increasing importance with increasing attention to the adverse effects of increased workload, especially in the medical field. Workload is generally considered to comprise the following two large aspects: objective workload that is simply reflected by the quantity of work tasks, and mental workload that reflects the mental strains resulting from a human operator performing a work task under a specific environmental or operational condition as well as the capability of the human operator to respond to task demands (16). Currently, the onset of modern technology and automation has greatly shifted the workload paradigm from the physical domain to the mental domain (16, 17), and some researchers thereby used mental workload as the measure of an individual's workload (18). Mental workload is also a term referring to the cost of completing a work task, and it can be defined as the amount of brain or cognitive resources used/consumed per unit time to reach the acceptable performance required by the work task (19). Currently, mental workload is widely accepted to be defined as the portion of a human operator's limited capacity actually required to perform a specific task under the assumption that humans have a fixed amount of processing capacity; and the task inherently requires processing resources, and the more difficult the task, the higher the processing capacity required for acceptable objective and subjective performance (16, 17).

#### Measurement of Workload

With respect to the workload measurement, the number of work tasks is often used to measure objective workload. For example, some single indicators such as working hours per week, and outpatient volume per shift (4, 11, 12) are used as the measure of physician workload in outpatient clinic. Such kind of the measurement of workload although simple and easy to be tracked and intervened in real time, might have failed to diagnose causes and determine the nature of workload, and explain the relation between the nature of a work task and the characteristics of the operator. In contrast, currently, there are three primary methods for measuring mental workload, mainly including subjective assessments, task-based performance measures, and physiological measures (16, 17, 20). Each of these methods can be applied in isolation, but they may be also measured concurrently to obtain a more comprehensive assessment of mental workload (16).

Subjective assessments concentrating on different aspects of mental workload require a human operator to distinguish a level of workload in indications on scales in post-task responses. The NASA-Task Load Index (NASA-TLX) scale, widely used in measuring or diagnosing mental workload in human factors and ergonomics, has proven to be a sensitive, valid and reliable assessment tool (21, 22), and can be used for quantifying perceived workload in various healthcare settings (23). In the same vein, the Subjective Workload Assessment Technology (SWAT) is a subjective rating technique used for assessing mental workload (24). Over the years, different researchers have localized these scales to be suitable for the measurement of mental workload in various settings in their own countries. For example, given that there are few instruments for measuring or diagnosing Chinese physicians' mental workload, our research team developed a mental workload scale verified with good reliability and validity based on the combination of dimensions of NASA-TLX and SWAT in 2018 (7). Although subjective measures of mental workload are with high face validity, ease of use, participant acceptability, low cost and known sensitivity to workload variation, there remain some methodological issues to be solved, such as trading off the intrusiveness of live ratings against the retrospective bias of post-task ratings (16, 17, 19). Accordingly, once a work task is finished, workload evaluation of the human operator is carried out immediately, possibly minimizing the recall bias.

Task-based performance measures are based on the performance variables (e.g., reaction times, accuracy, and error rates) of a human operator in performing work tasks as assessment indicators of mental workload under an underlying assumption that increased processing required for higher levels of workload would degrade the performance of the task being performed, mainly including primary and secondary task measures (17). Performance is considered as a measure of primary and secondary task achievement and can indicate spare capacity. Primary task measure is based on techniques of direct registration of the human operator's capability to perform the primary task at an acceptable level in the natural or simulated work environment (for example, with respect to acceptably low error likelihood), whereas in secondary task measures, monitoring attention to and workload resulting from a primary task may be indirectly reflected by assessing the performance on a secondary task, since in any real-world dual task situation where one task takes priority over the other, the performance on the secondary task (e.g., errors and time) can be highly associated with the spare capacity unused by the primary task (17). A primary task analysis is the most fundamental means of assessing mental workload using these measures (16). Task analysis includes any methods of evaluating what actions a human operator performs and why these actions are being performed based on a time-motion study; and these methods involve the structured decomposition of work activities and classification of these activities as a series of tasks, processes, or classes (17). However, the insensitivity of some of the measurements based on these performance-based techniques is one disadvantage, since there is not a simple linear, but an inverted U relationship between mental workload and job performance. That is, a work task with a low demanding mental workload could achieve an excellent performance during the beginning stage of the task, but then the performance will be degraded as the human operator becomes fatigued or distracted, possibly leading to confounded results (19). Accordingly, using task performance techniques with other mental workload assessment methods may improve the quality of the measurement (19).

Physiological measures are the third type of mental workload assessments, as a natural type of mental workload index, under the underlying assumption that changes of mental workload level of a human operator can be reflected by the changes in some physiological indicators of the human operator corresponding to different task demands. Previous studies have used physiological parameters such as heart rate, heart rate variability, brain activity, event-related brain potential, galvanic skin resistance, breathing rate, pupil diameter and blink rates to assess the human operator's state (16, 17, 25). Although measuring physiological parameters for mental workload evaluation has been fairly well-validated with decreased intrusiveness, much of the research done involves a controlled experiment with controlled stimuli, since on the one hand, many other factors that have no relation to mental workload, might also cause changes in certain physiological parameters (15), and on the other hand, the accuracy of physiological data depends to a large extent on the performance and precision of the sensors (19). In addition to these above-mentioned measures for physician workload, workload was frequently tracked as relative value units (26).

#### Physician Work Tasks Tethered to Outpatient Practice

Identifying tasks refines the measurement of workload associated with these tasks. Work tasks tethered to outpatient practice can be generalized as the sum of a series of work activities necessary for physicians to provide outpatients with complete medical services that meet their reasonable demands. In our previous study, we decomposed and further classified all work activities physicians performed during outpatient encounters into the two large groups based on a time-motion task analysis on 32 Chinese physicians from public tertiary hospitals: physician-patient communication work tasks (comprising six categories of work tasks) characterized by direct patient interaction, and non-physician-patient communication work tasks (comprising seven categories of work tasks) characterized by paperwork (27). Research indicated that gaining insight into the effort associated with work tasks could make workload assessments more robust, since task analysis might facilitate workload measurement by the individual work components or subtasks to be measured (28). The definition of the detailed work tasks performed by physicians in outpatient practice can provide a solid foundation for the present study to further seek to comprehensively assess physician workload with the consideration of the content and nature of work tasks tethered to outpatient practice.

#### Evaluation Framework for Physician Workload

Previous studies have investigated the physician workload in various working settings [e.g., emergency department (16, 23), clinical care (18, 25)] using single methods noted in the literature review or a combination of these methods (16); however, most of them might have failed to comprehensively assess physician workload, since they either only focused on the objective workload (4) or mental workload using single or multiple metrics, or just simply measured both objective workload and mental workload to obtain a more comprehensive assessment of physician workload (16). It is obvious that such a single or non-integrated evaluation framework will not effectively solve comprehensive assessments of physician workload. To this end, some research has developed and applied a research model of factors affecting attending physician workload centered on physician, hospital, team and patient characteristics through in-depth semi-structured interviews and a modified Delphi technique (26, 29).

Workload is also influenced in part by the nature of the work task in question (28). Research indicated that physician workload is increased with complexity of medical cases or medical care (16, 18, 23, 26, 30, 31), and the well-known RBRVS (resource-based relative value scale, RBRVS) model, widely used for medical workers' performance appraisal, revealed that these patient factors such as complexity, and severity of the patient's disease had a positive impact on the physician workload. Thus, patient complexity should be considered into the comprehensive assessment framework of physician workload. However, there has little research into the application of patient complexity as an adjustment coefficient to adjust the physician workload. Moreover, research also revealed that practice size and quality of medical services were key factors influencing physician workload (23, 26, 32); and physician workload is increased with number of patients seen (18, 23, 33), and the higher the quality of medical care provided by physicians, the more their resources are consumed, ultimately resulting in a higher workload (34), whereas in turn, increased workload might reduce medical quality (5, 7–9). Thus, practice size and quality of medical services should be also considered. Moreover, there has little research into assessment of physicians' outpatient workload at task level based on the content of work tasks performed (35) in outpatient settings. Some studies addressed the assessment of physician workload at task level (named task-level workload in the rest of the paper) in emergency department (16), internal medicine (28), clinical radiation oncology (36, 37), and general practice (38). Such kind of study provides a reasonable method to quantify physician workload for detailed activities, which may be useful to monitor workload for more granular tasks within activities.

**Figure 1:**
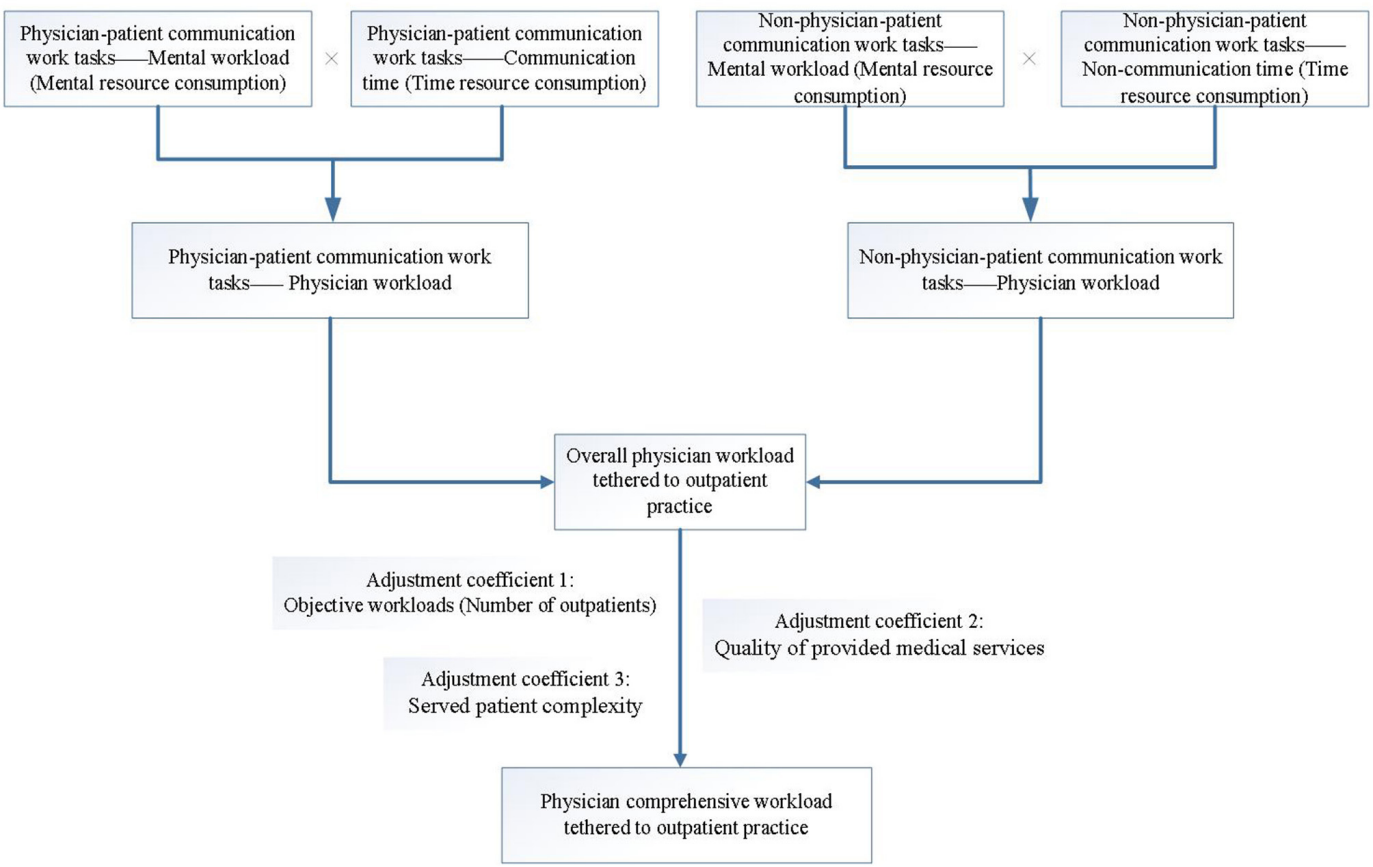
Comprehensive evaluation framework for physician workload tethered to outpatient practice.

When drawing insights from previous studies on assessments of physician workload, we proposed a comprehensive evaluation framework for physician workload based on the combination of objective workload and task-level mental workload also with the consideration of quality of provided medical services and served patient complexity (see Figure 1). This evaluation framework guided our study to develop an integrated evaluation model for physician comprehensive workload tethered to outpatient practice, and further perform a physician comprehensive workload analysis among Chinese physicians.

## Methods

Through this paper, we performed a physician comprehensive workload analysis among Chinese physicians according to our proposed comprehensive evaluation framework for physician workload tethered to outpatient practice using cross-sectional data on outpatient physician workload from a nationwide survey.

### Calculation Procedure of Comprehensive Physician Workload

According to our proposed comprehensive evaluation framework for physician workload tethered to outpatient practice, we further developed an integrated evaluation model for physician comprehensive workload (*PCW*) tethered to outpatient practice, where PCW comprises two parts (that is, physician workload tethered to physician-patient communication work tasks, and physician workload tethered to non-physician-patient communication work tasks), and is further adjusted together by objective workload, quality of provided medical services, and served patient complexity (Equation 1).


(1) $$\begin{array}{l} PCW_{i}=\left({PW_{Communication}}_{i}+{PW_{Non-communication}}_{i}\right)\\ \;*\;{R_{ov}}_{i}\;*\;{R_{pc}}_{i}\;*\;{R_{ps}}_{i} \end{array}$$

*PCW*_*i*_ is the value of physician comprehensive workload tethered to outpatient practice of the *i*th physician, *PW*_*Communication*_*i*__ is the value of physician workload of the *i*th physician while performing physician-patient communication work tasks, *PW*_*Non*−*communication*_*i*__ is the value of physician workload of the *i*th physician while performing non-physician-patient communication work tasks; and *R*_*ov*_, *R*_*pc*_, and *R*_*ps*_ are used as adjustment coefficients for *PCW*; and therein *R*_*ovi*_ represents the objective workload of the *i*th physician, reflected by the number of outpatients serviced per day of the *i*th physician, *R*_*pc*_*i*__ represents the complexity of the patient served by the *i*th physician in the diagnosis and treatment, reflected by the ratio of the number of outpatients serviced per day admitted to the hospital by the *i*th physician for further diagnosis or treatment to the number of outpatients seen per day by the *i*th physician, and *R*_*ps*_*i*__ represents the quality of medical services provided by the *i*th physician, reflected by the patient satisfaction rated by the *i*th physician in this study. Given that mental workload can be defined as the amount of mental resources used/consumed per unit time to reach the acceptable performance required by the work task (19), representing the occupancy rate of an individual's mental resources on the work task, physician workload tethered to a specific work task can be therefore defined as the mental resources consumed by the work task multiplied by the time resources required by the work task (Equation 2, 3).


(2) $$\begin{array}{l} {PW_{Communication}}_{i}\;=\;{PMW_{Communication}}_{i}\;*\;{T_{Communication}}_{i} \end{array}$$

*PMW*_*Communication*_*i*__ is the value of mental workload of the *i*th physician, reflecting mental resource consumption while performing physician-patient communication work tasks, whereas *T*_*Communication*_*i*__ represents the time resources required by the physician-patient communication work tasks performed by the *i*th physician.


(3) $$\begin{array}{l} {PW_{Non-communication}}_{i}={PMW_{Non-communication}}_{i}\\ \;*\;{T_{Non-communication}}_{i} \end{array}$$

Likewise, *PW*_*Non*−*communication*_*i*__ is the value of physician workload of the *i*th physician, reflecting mental resource consumption while performing non-physician-patient communication work tasks, whereas *T*_*Non*−*communication*_*i*__ represents the time resources required by the non-physician-patient communication work tasks performed by the *i*th physician.

### Measures

Mental workload was assessed using the Chinese version of physician mental workload scale developed by our research team in 2018 based on the combination of dimensions of the NASA-TLX and SWAT frameworks (7). The Chinese version of physician mental workload scale has been verified to have good reliability and validity (Cronbach alpha = 0.81), indicating a reliable instrument for diagnosing the mental workload of Chinese physicians comprises six dimensions and 12 items regarding different aspects of workload (mental demands, MD; physical demands, PD; temporal demands, TD; perceived risk, PR; frustration level, FL; and performance, Pe) (7); and all items were scored on a 10-point bipolar scale ranging from 0 to 100. The average score of all items of a corresponding dimension was the dimension score, whereas each dimension score was multiplied by the weight of the corresponding dimension, and the sum of the scores was the total score of mental workload, where the weight of each dimension was equal to the number of times that dimension was selected divided by a total of 15 comparisons based on pairwise comparisons of six dimensions of physician mental workload scale (7). In this study, the Chinese version of physician mental workload scale was used to measure physician mental workload while performing physician-patient communication work tasks (Equation 4) and non-physician-patient communication work tasks, respectively (Equation 5).


(4) $$\begin{array}{l} {PMW_{Communication}}_{i}=\;MD_{i}\;*\;W_{MDi}+PD_{i}\;*\;W_{PDi}+TD_{i}\;*\;W_{TDi}\\ \;+PR_{i}\;*\;W_{PRi}+FL_{i}\;*\;W_{FLi}+Pe_{i}\;*\;W_{Pei} \end{array}$$

*PMW*_*Communication*_*i*__ is the overall mental workload score of the *i*th physician while performing physician-patient communication work task, and *MD*_*i*_, *PD*_*i*_, *TD*_*i*_, *PR*_*i*_, *FL*_*i*_, and *Pe*_*i*_ is the average score of each dimension the *i*th physician rated, respectively, and *W* with a subscript is the weighting coefficient of the corresponding dimension of the *i*th physician.


(5) $$\begin{array}{l} {PMW_{Non-communication}}_{i}=\;MD_{i}\;*\;W_{MDi}+PD_{i}\;*\;W_{PDi}\\ \;+TD_{i}\;*\;W_{TDi}+PR_{i}\;*\;W_{PRi}\\ \;+FL_{i}\;*\;W_{FLi}+Pe_{i}\;*\;W_{Pei} \end{array}$$

*PW*_*Non*−*communication*_*i*__ is the overall mental workload score of the *i*th physician while performing non-physician-patient communication work tasks, and likewise, *MD*_*i*_, *PD*_*i*_, *TD*_*i*_, *PR*_*i*_, *FL*_*i*_, and *Pe*_*i*_ is the average score of each dimension the *i*th physician rated, respectively, and *W* with a subscript is the weighting coefficient of the corresponding dimension of the *i*th physician.

For objective workload, we used the number of outpatients serviced per day as the measure in outpatient practice, since it can better reflect the quantity of work tasks associated with patients than other indicators, such as outpatient working hours. For quality of provided medical services, previous research indicated that patient satisfaction is an important indicator of measuring the quality of medical services (39), and therefore was simply used as a measure of quality of provided medical services in this study.

Given that it is difficult to quantity the complexity of the patient serviced by physicians in outpatient clinics in the diagnosis and treatment also with no accepted measures, patient difficulty served by a physician in the diagnosis and treatment could be simply measured based on the question that “did you have any patients that you perceived as difficult?” reported in previous research (23) and another study reported some domains such as number of major comorbidities, major complications, and operation could be used as a measure of patient complexity by surgical specialty (40). And accordingly, we used the ratio of the number of outpatients serviced per day admitted to the hospital by a physician for further diagnosis or treatment to the number of outpatients seen per day by the physician as the measure of served patient complexity in this study; and to avoid that the ratio was zero, we normalized this ratio and added 1, that is, 1 represents the reference value. For time resource consumption, time resource consumption in physician-patient communication work tasks was measured by the communication time during each outpatient encounter, and likewise, time resource consumed on non-physician-patient communication work tasks was measured by the non-communication time during each outpatient encounter.

### Questionnaire Design

According to our proposed integrated evaluation model for physician workload, based on the Chinese version of physician mental workload scale developed by our research team in 2018, we therefore developed a survey questionnaire to perform a PCW analysis among Chinese physicians.

The survey questionnaire included three parts. The first part was designed to measure the mental workload of physicians while performing physician-patient communication work tasks in outpatient practice, including 6 dimensions and 12 items of Chinese version of physician mental workload scale, where these dimensions were further compared two by two making able to collect the weights of each dimension. Likewise, the second part was designed to assess the mental workload of physicians while performing non-physician-patient communication work tasks in outpatient practice using the Chinese version of physician mental workload scale. Data on participants' characteristics including gender, age, marital status, educational level, average monthly income and professional title, average monthly income, professional title, working years in the current medical institution, area, hospital level, hospital nature, personnel, department, working hours per week, outpatient working hours per week, self-rated health status and physician-patient communication time and non-physician-patient communication time, as well as these related adjustment coefficients for *PCW* (that is, number of outpatients serviced per day, outpatient satisfaction, and number of outpatients serviced per day admitted to the hospital for further diagnosis or treatment) (details seen from Table 1) were collected in the third part.

**Table 1:** Main characteristics of the participating physicians (*N* = 1,934).

**Characteristics**	***N* (%)**	**Characteristics**	***N* (%)**
Gender		Area	
Male	1,047 (54.1)	Eastern China	735 (38.0)
Female	887 (45.9)	Central China	685 (35.4)
Age (years)		Western China	514 (26.6)
20–30	433 (22.4)	Hospital level	
31–40	852 (44.1)	Tertiary A hospital	1,234 (63.8)
41–55	587 (30.4)	Tertiary B hospital	215 (11.1)
>55	62 (3.2)	Secondary hospital	447 (23.1)
Marital status		First-tier hospital	38 (2.0)
Unmarried	305 (15.8)	Department	
Married	1,585 (82.0)	Internal	585 (30.2)
Divorced	36 (1.9)	Surgical	481 (24.9)
Widowed	8 (0.4)	Obstetrics and gynecology	192 (9.9)
Educational level		Pediatrics	163 (8.4)
Doctorate	228 (11.8)	Others	513 (26.5)
Master	776 (40.1)	Working hours per week	
Undergraduate	857 (44.3)	≤ 40	180 (9.3)
Junior college	59 (3.1)	41–60	1062 (54.9)
Others	14 (0.7)	>60	692 (35.8)
Professional title		Outpatient working hours per week	
Senior	212 (11.0)	≤8	584 (30.2)
Associate senior	548 (28.3)	~16	440 (22.8)
Intermediate	699 (36.1)	~24	440 (22.8)
Junior	450 (23.3)	~40	268 (13.9)
Others	25 (1.3)	>40	202 (10.4)
Working years in the current medical institution		Self-rated health status	
1–5	596 (30.8)	Very poor	23 (1.2)
6–10	503 (26.0)	Poor	105 (5.4)
11–15	335 (17.3)	Fair	902 (46.6)
16–20	206 (10.7)	Good	624 (32.3)
>20	294 (15.2)	Very good	280 (14.5)
Hospital nature		Number of outpatients serviced per day (Mean ± SD)	43.20 ± 24.81
Public general hospital	1,812 (93.7)	Number of outpatients serviced per day admitted to the hospital (Mean ± SD)	4.22 ± 3.85
Public specialized hospital	98 (5.1)	Self-rated outpatient satisfaction (Mean ± SD)	80.59 ± 14.75
Private general hospital	11 (0.6)	Physician-patient communication time per visit (Mean ± SD/minutes)	6.02 ± 3.89
Private specialized hospital	13 (0.7)	Non-physician-patient communication time per visit (Mean ± SD/minutes)	3.95 ± 3.30

Specifically, outpatient satisfaction was measured based on the question on a 20-point bipolar scale ranging from 0 to 100, that is, “Overall, how many scores did you perceive that your outpatients rate for your outpatient services?”. The number of outpatients serviced per day was measured based on the question that “on average, how many outpatients did you serve daily?”, and the number of outpatients serviced per day admitted to the hospital for further diagnosis or treatment was further measured based on the question that “how many outpatients you serviced were admitted to the hospital for further diagnosis or treatment per day?” Physician-patient communication time was measured based on the question that “how many minutes do you spend on physician-patient communication work tasks on average during each outpatient encounter?”, and likewise, non-physician-patient communication time was measured based on the question that “how many minutes do you spend on non-physician-patient communication work tasks on average during each outpatient encounter?”.

Before the formal survey began, we performed a pilot survey on site in October 2020, to validate the measurement tool in a total of 10 physicians who just finished the provision of the outpatient services in the outpatient clinic of a tertiary public hospital in Wuhan, Hubei, and context-specific adjustments were made to improve the accuracy and clarity of the questionnaire according to the feedback from the pilot survey.

### Study Sampling

This cross-sectional nationwide survey recruited physicians in Eastern, Central, and Western China through a stratified convenience sampling method. In order to improve the sample representativeness, two provinces were selected in the Eastern, Central, and Western regions, respectively. That is, a total of six provinces were selected. According to the standard for the division of China Eastern, Central, and Western regions from the current China Health Statistics Yearbook, Guangdong and Zhejiang provinces were selected in Eastern China, Hubei and Henan provinces were selected in Central China, and Chongqing municipality and Guangxi Zhuang autonomous region were selected in Western China. Considering the potential differences in the different levels of hospitals, typical sampling was then used to select two tertiary public hospitals and two secondary public hospitals in each selected province. Thus, a total of 24 public hospitals (including 12 tertiary and 12 secondary public hospitals) were mainly selected nationwide in China. Among the selected hospitals, four departments including Internal, Surgical, Obstetrics and gynecology, and Pediatrics were further selected as the main research departments, where targeted physicians were selected by random sampling. Given that we aimed to assess the physician workload tethered to outpatient practice, the targeted population in this nationwide survey was physicians who just provided medical services to outpatients in the consulting room in outpatient clinics, those who had to have been working for ≥4 months in the outpatient clinic, and those who had to be employed full-time for ≥1 year in their current institution.

### Data Collection

This nationwide survey was carried out from November, 2020 to February, 2021. In order to improve the efficiency of data collection in each selected hospital, a unique QR code of the electronic questionnaire was generated for each hospital using the web-based survey tool called wenjuanxing. An informed consent of the outpatient managers in each selected hospital was requested and obtained before the beginning of the survey, and they were invited and volunteered as the role of the project manager in their hospitals during this online survey. Subsequently, the QR code of the electronic questionnaire was sent to these outpatient managers of the corresponding hospital, and they then sent the QR code to the targeted department groups of physicians via WeChat or Tencent QQ group, in which physicians who met the inclusion criteria for the targeted population were invited to participate in this questionnaire survey.

Participants could scan the QR code of the electronic questionnaire via their phones to access and fill in the electronic questionnaire. Prior to the survey, we first introduced the purpose of the survey, and the definition of physician mental workload and its related work tasks including physician-patient communication and non-physician-patient communication work tasks during outpatient encounters. After an individual's consent was obtained, the survey was conducted accordingly. During filling in the questionnaire, participants were first asked to complete the assessment of mental workload while performing physician-patient communication work tasks, and were then allowed to report their mental workload while performing non-physician-patient communication work tasks. Furthermore, all participants who completed the questionnaire were also encouraged to share the survey website link to their Wechat Circle of Friends, WeChat or Tencent QQ group, where some physicians who met the inclusion criteria for the targeted population could be invited and participate in this questionnaire survey.

### Statistical Analysis

Descriptive analysis was used to summarize to data on participant characteristics, and PCW of the participating physicians. Data were summarized as frequencies (*n*) and percentages (%) for categorical variables, and mean and standard deviation (M ± SD) for continuous variables.

There is still lack of consensus on what should be considered as a threshold value for a high or excessive workload internationally, and previous research usually classified individuals with high workload among physicians simply using the quartiles (35, 41), threshold values for workload (50% of overall workload) (42) or high workload corresponding to composite NASA-TLX scores of >55 (43) or >60 (44). To discriminate the relative comprehensive workload level among the participating physicians, the PCW was divided into four different levels using *M*−*SD*, *M* and *M*+*SD* as cut points based on the measurement results of PCW, namely I-type (*PCW* ≤ *M*−*SD*), II-type (*M*−*SD* < *PCW* ≤ *M*), III-type (*M* < *PCW* ≤ *M*+*SD*) and IV-type (*PCW* > *M*+*SD*), respectively, which can be distinguished as having relative low (low PCW physicians), medium (medium PCW physicians), high (high PCW physicians) and very high (very high PCW physicians) levels of PCW. Therefore, four distinctive groups of PCW physicians were identified, and each participating physician was assigned into one of the PCW physician groups.

Multiple linear regression model was established to identify the significant factors that influenced PCW tethered to outpatient practice. Given that data on PCW did not follow a normal distribution, we therefore converted the PCW into ln(*PCW*) to meet the normal distribution, before the regression to improve the accuracy of parameters estimation. Previous research revealed that individual characteristics were the key factors that influenced mental workload (45), and therefore, all demographic variables were set as independent variables and included in the model.

Moreover, chi-square (χ^2^) tests were then performed to explore the differences in the four distinctive groups of PCW physicians across characteristics; and multinomial logistic regression was further used to identify the significant predictors of four groups of PCW physicians regarding their comprehensive workload, and therein the variables on characteristics were set as independent variables. Data analyses were performed using STATA 15.0 software. *P* < 0.10 (optimally, *p* < 0.05) was considered statistically significant.

## Results

### Participant Characteristics

Overall, 2,038 online responses were collected and 1,934 eligible responses were received, with an effective recovery rate of 94.9%. Table 1 shows the detailed characteristics of the 1,934 samples. Of these participating physicians, 54.1% were female, 44.1% were aged 31 to 40 years, 63.8% were from tertiary A hospitals, 38.0% were from Eastern China, and most were from the 4 major departments (Internal, Surgical, Obstetrics and gynecology, and Pediatrics), which accounted for 73.5%. The participating physicians serviced an average of 43.20 (SD = 24.81) patients per day in outpatient clinics and therein reported an average of 4.22 (SD = 3.85) patients admitted into the hospital for further diagnosis or treatment. Moreover, the mean score of outpatient satisfaction rated by physicians was 80.59 (SD = 14.75).

### Assessments of Physician Comprehensive Workload Tethered to Outpatient Practice

According to our developed integrated evaluation model for physician workload proposed in this study, measurement results showed that the mean score of PCW tethered to outpatient practice among the 1,934 participating physicians was 811.30 (SD = 494.98), ranging from 68.87 to 5399.20. Table 2 further shows the number of participating physicians at different levels of PCW scores, with concentrating on between 200 and 1,200. Among these participating physicians, 14.7% (*n* = 284) scored between 200 and 400, 19.5% (*n* = 377) scored between 400 and 600, 18.5% (*n* = 358) scored between 600 and 800, 14.9% (*n* = 288) scored between 800 and 100, and 12.5% (*n* = 241) scored between 1,000 and 1,200, together accounting for 80.1%, whereas only 6.9% (*n* = 134) scored between 1,200 and 1,400, and <10% (9.4%; *n* = 182) scored more than 1,400. Moreover, 42.9% (*n* = 830) scored higher than the average score of the total sample.

**Table 2:** Distribution of participating physicians at different levels of scores of physician comprehensive workload tethered to outpatient practice (*N* = 1,934).

**Physician comprehensive workload**	***N***	**Proportion (%)**	**Cumulative proportion (%)**
(0,200]	70	3.6	3.6
(200,400]	284	14.7	18.3
(400,600]	377	19.5	37.8
(600,800]	358	18.5	56.3
(800,1,000]	288	14.9	71.2
(1,000,1,200]	241	12.5	83.7
(1,200,1,400]	134	6.9	90.6
(1,400,1,600]	77	4.0	94.6
(1,600,1,800]	40	2.0	96.6
(1,800,5,400)	65	3.4	100.0

### Discrimination of Relative Physician Comprehensive Workload Level and Its Characteristics

To discriminate the relative comprehensive workload level among the participating physicians, the PCW was divided into four different levels using *M*−*SD*, *M* and *M*+*SD* as cut points based on the measurement results of PCW, namely I-type (*PCW* ≤ 316.32), II-type (316.32 < *PCW* ≤ 811.30), III-type (811.30 < *PCW* ≤ 1306.28), and IV-type (*PCW* > 1306.28), respectively, which can therefore be distinguished as having relative low (low PCW physicians), medium (medium PCW physicians), high (high PCW physicians) and very high (very high PCW physicians) levels of PCW. Therefore, four distinctive groups of PCW physicians were identified; and of these participating physicians, 11.2% (*n* = 236) were classified as very high PCW physicians, compared with 11.6% as low PCW physicians, 45.5% as medium PCW physicians and 30.7% as high PCW physicians.

Table 3 further presents the significant characteristics of the participating physicians at different levels of PCW tethered to outpatient practice. Chi-square tests indicated that there was a significant difference in the four levels of PCW for gender (χ^2^ = 36.523, *p* < 0.001), educational level (χ^2^ = 22.135, *p* = 0.008), average monthly income (χ^2^ = 18.878, *p* = 0.026), professional title (χ^2^ = 28.729, *p* = 0.001), working years in the current medical institution (χ^2^ = 23.979, *p* = 0.020), area (χ^2^ = 37.058, *p* < 0.001), hospital level (χ^2^ = 40.095, *p* < 0.001), department (χ^2^ = 75.682, *p* < 0.001), working hours per week (χ^2^ = 16.088, *p* = 0.013), and outpatient working hours per week (χ^2^ = 43.052, *p* < 0.001).

**Table 3:** Significant characteristics of participating physicians with different levels of comprehensive workload tethered to outpatient practice (*N* = 1,934).

**Characteristics (*N*, %)**	**Low PCW physician (*N* = 224)**	**Medium PCW physician (*N* = 880)**	**High PCW physician (*N* = 594)**	**Very high PCW physician (*N* = 236)**	**χ^2^**	***p*-value**
Gender					36.523	<0.001
Male	135 (60.3)	527 (59.9)	283 (47.6)	102 (43.2)		
Female	89(39.7)	353 (40.1)	311 (52.4)	134 (56.8)		
Educational level					22.135	0.008
Doctorate	17 (7.6)	120 (13.6)	66 (11.1)	25 (10.6)		
Master	81 (36.2)	358 (40.7)	260(43.8)	77 (32.6)		
Undergraduate	116 (51.8)	370 (42.0)	250 (42.1)	121 (51.3)		
Junior college	7 (3.1)	25 (2.8)	16 (2.7)	11 (4.7)		
Others	3 (1.3)	7 (0.8)	2 (0.3)	2 (0.8)		
Average monthly income (RMB)					18.878	0.026
≤5,000	48 (21.4)	181 (20.6)	98 (16.5)	49 (20.8)		
5,001–10,000	122 (54.5)	383 (43.9)	283 (47.6)	112 (47.5)		
10,001–15,000	37 (16.5)	185 (21.0)	137 (23.1)	47 (19.9)		
>15,000	17 (7.6)	128 (14.5)	76 (12.8)	28 (11.9)		
Professional title					28.729	0.001
Senior	15 (6.7)	116 (13.2)	57 (9.6)	24 (10.2)		
Associate senior	58 (25.9)	226 (25.7)	208 (35.0)	56 (23.7)		
Intermediate	87 (35.9)	312 (35.5)	206 (34.7)	94 (39.8)		
Junior	61 (27.2)	209 (23.8)	120 (20.2)	60 (25.4)		
Others	3 (1.3)	17 (1.9)	3 (0.5)	2 (0.8)		
Working years in the current medical institution					23.979	0.020
1–5	78 (34.8)	283 (32.2)	168 (28.3)	67 (28.4)		
6–10	58 (25.9)	217 (24.7)	179 (30.1)	49 (20.8)		
11–15	38 (17.0)	149 (16.9)	101 (17.0)	47 (19.9)		
16–20	23 (10.3)	81 (9.2)	64 (10.8)	38 (16.1)		
>20	27 (12.1)	150 (17.0)	82(13.8)	35 (14.8)		
Area					37.058	<0.001
Eastern China	64 (28.6)	358 (40.7)	243 (40.9)	70 (29.7)		
Central China	108 (48.2)	305(34.7)	194 (32.7)	78 (33.1)		
Western China	52 (23.2)	217 (24.7)	157 (26.4)	88 (37.3)		
Hospital level					40.095	<0.001
Tertiary A hospital	118(52.7)	595 (67.6)	375 (63.1)	146 (61.9)		
Tertiary B hospital	28 (12.5)	83 (9.4)	91 (15.3)	13 (5.5)		
Secondary hospital	66 (29.5)	184 (20.9)	122 (20.5)	75 (31.8)		
First-tier hospital	12 (5.4)	18 (2.0)	6 (1.0)	2 (0.8)		
Department					75.682	<0.001
Internal	66 (29.5)	260 (29.5)	188 (31.6)	71(30.1)		
Surgical	61 (27.2)	265 (30.1)	102 (17.2)	53 (22.5)		
Obstetrics and Gynecology	19 (8.5)	64 (7.3)	96 (16.2)	13 (5.5)		
Pediatrics	21 (9.4)	72 (8.2)	58 (9.8)	12 (5.1)		
Others	57 (25.4)	219 (24.9)	150 (25.3)	87 (36.9)		
Working hours per week					16.088	0.013
≤40	28 (12.5)	89 (10.1)	35 (5.9)	28 (11.9)		
41–60	124 (55.4)	475 (54.0)	346 (58.2)	117 (49.6)		
>60	72 (32.1)	316 (35.9)	213 (35.9)	91 (38.6)		
Outpatient working hours per week					43.052	<0.001
≤8	79 (35.3)	301 (34.2)	155 (26.1)	49 (20.8)		
~16	51 (22.8)	210 (23.9)	127 (21.4)	52 (22.0)		
~24	47 (21.0)	179 (10.3)	158 (26.6)	56 (23.7)		
~40	34 (15.2)	110 (12.5)	84 (14.1)	40 (16.9)		
>40	13 (5.8)	80 (9.1)	70 (11.8)	39 (16.5)		

When compared with those classified as the other groups, physicians classified as very high PCW physician group, tended to be those who were female, those who had a lower educational level, lower average monthly incomes, lower professional titles and longer working years in the current medical institution, and those who were from Western China, worked in tertiary A hospitals, and Internal or Surgical, and worked no more than 40 h per week and longer outpatient hours per week.

### Factors Associated With Physician Comprehensive Workload

Table 4 presents the results derived from multiple linear regression analysis. The model included all independent variables on characteristics, and there existed only four significant variables in the model.

**Table 4:** Factors associated with the physician comprehensive workload (*N* = 1,934; df = 1,886).

**Independent variables**	**β**	**SE**	***t*-test**	***p*-value**	**VIF**
Constant	6.353	0.103	61.67	<0.001	
**Gender (ref: Male)**					
Female	0.115	0.0336	3.41	0.001	1.38
**Area (ref: Central China)**					
Eastern China	0.104	0.0356	2.92	0.004	1.60
Western China	0.134	0.0400	3.36	0.001	1.53
**Working hours per week (ref: >60)**					
≤40	−0.156	0.601	−2.59	0.010	1.36
41–60	−0.0817	0.0313	−2.61	0.009	1.30
**Outpatient working hours per week (ref: ≤8)**					
~16	0.0372	0.0383	0.97	0.331	1.42
~24	0.123	0.0415	2.97	0.003	1.50
~40	0.129	0.0505	2.56	0.010	1.48
>40	0.250	0.0513	4.89	<0.001	1.38

*SE, standard error; VIF, variance inflation factor (mean VIF = 1.87, ranging from 1.15–4.78)*.

Gender, area, working hours per week, and outpatient working hours per week significantly (*p* < 0.05) influenced PCW tethered to outpatient practice. Females experienced a higher PCW in outpatient practice than males (β= 0.115, *p* = 0.001). For area, physicians from Eastern or Western China experienced a higher PCW than those from Central China (β= 0.104, *p* = 0.004; β= 0.134, *p* = 0.001, respectively). For working hours per week, compared to those who worked >60 h per week, physicians with shorter working hours per week had a lower PCW in outpatient practice (β= −0.156, *p* = 0.010; β= −0.0817, *p* = 0.009, respectively). Meanwhile, physicians with longer outpatient working hours per week tended to experience a higher PCW in outpatient practice; compared to those who worked no more than 8 h per week, physicians with longer working hours in outpatient practice had a higher PCW (β= 0.123, *p* = 0.003; β= 0.129, *p* = 0.010, β= 0.250, *p* < 0.001; respectively). Moreover, the results of the variance inflation factor (VIF) showed that all values of the VIF ranged from 1.15 to 4.78, indicating that there was no collinearity among these independent variables included in the model.

### Determinants of Relative Levels of Physician Comprehensive Workload

Multinomial logistic regression was further used to identify the significant predictors of the physicians belonging to different groups of PCW physicians. Table 5 shows the results of multinomial logistic regression analysis. Using very high PCW physician group as the base outcome, we gained the following results (Table 5).

**Table 5:** Multinomial logistic regression result: significant determinants of different groups of PCW physicians.

	**Low PCW physician**		**Medium PCW physician**		**High PCW physician**	
**Variables**	**RRR (95% CI)**	***p***	**RRR (95% CI)**	***p***	**RRR (95% CI)**	***p***
**Gender (ref: Male)**						
Female	0.370 (0.236,0.580)	<0.001^***^	0.427 (0.299,0.609)	<0.001^***^	0.509 (0.351,0.739)	<0.001^***^
**Age (ref: 20–30 years)**						
31–40	0.901 (0.436,1.860)	0.778	0.969 (0.531,1.768)	0.917	0.506 (0.269,0.952)	0.035^**^
41–55	0.529 (0.209,1.341)	0.180	0.883 (0.414,1.881)	0.747	0.392 (0.178,0.865)	0.020^**^
>55	0.439 (0.108,1.793)	0.251	0.440 (0.144,1.341)	0.149	0.323 (0.101,1.032)	0.057^*^
**Educational level (ref: Master)**						
Doctorate	0.700 (0.319,1.537)	0.374	0.900 (0.511,1.585)	0.715	0.751 (0.412,1.366)	0.348
Undergraduate	0.784 (0.480,1.283)	0.333	0.653 (0.445,0.959)	0.030^**^	0.690 (0.461,1.032)	0.071^*^
Junior college	0.380 (0.112,1.292)	0.121	0.456 (0.184,1.129)	0.089^*^	0.685 (0.263,1.783)	0.438
Others	N/A	N/A	N/A	N/A	N/A	N/A
**Professional title (ref: Junior title)**						
Senior	1.310 (0.427,4.020)	0.636	1.713 (0.731,4.016)	0.216	2.564 (1.028,6.393)	0.043^**^
Associate senior	2.119 (0.917,4.896)	0.079^*^	1.864 (0.945,3.677)	0.072^*^	3.903 (1.895,8.041)	<0.001^***^
Intermediate	1.092 (0.561,2.125)	0.797	1.101 (0.639,1.895)	0.729	1.617 (0.901,2.903)	0.107
Others	N/A	N/A	N/A	N/A	N/A	N/A
**Working years in the current medical institution (ref: 6–10 years)**						
1–5	1.064 (0.569,1.988)	0.847	0.951(0.568,1.591)	0.847	0.728 (0.424,1.252)	0.252
11–15	0.682 (0.365,1.274)	0.230	0.627 (0.383,1.026)	0.063^*^	0.526 (0.314,0.881)	0.015^**^
16–20	0.520 (0.233,1.162)	0.111	0.388 (0.207,0.728)	0.003^***^	0.367 (0.191,705)	0.003^***^
>20	0.889 (0.360,2.198)	0.800	0.886 (0.444,1.768)	0.731	0.609 (0.295,1.259)	0.181
**Area (ref: Western China)**						
Eastern China	1.095 (0.641,1.873)	0.739	1.573 (1.051,2.354)	0.028^**^	1.677 (1.099,2.558)	0.016^**^
Central China	1.957 (1.177,3.254)	0.010^**^	1.627 (1.090,2.430)	0.017^**^	1.457 (0.955,2.222)	0.081^*^
**Hospital level (ref: Tertiary A hospital)**						
Tertiary B hospital	2.075 (0.931,4.621)	0.074^*^	1.437 (0.720,2.869)	0.304	1.987 (0.981,4.024)	0.057^*^
Secondary hospital	1.278 (0768,2.129)	0.345	0.846 (0.564,1.268)	0.418	0.824 (0.536,1.265)	0.043^**^
First-tier hospital	N/A	N/A	N/A	N/A	N/A	N/A
**Department (ref: Pediatrics)**						
Internal	0.451 (0.196,1.042)	0.062^*^	0.490 (246,0.987)	0.046^***^	0.482 (0.234,0.987)	0.046^**^
Surgical	0.388 (0.160,0.940)	0.036^**^	0.433 (0.206,0.908)	0.027^**^	0.227 (0.227,0.491)	<0.001^***^
Obstetrics and Gynecology	0.868 (0.299,2.523)	0.796	0.816 (0.334,1.993)	0.656	1.170 (0.477,2.869)	0.731
Others	0.338 (0.147,0.779)	0.011^**^	0.351 (0.175,0.703)	0.003^***^	0.330 (0.162,0.672)	0.002^***^
**Working hours per week (ref: 41–60)**						
≤40	0.815 (0.419,1.585)	0.547	0.775 (0.453,1.327)	0.353	0.439 (0.240,0.802)	0.007^***^
>60	0.585 (0.376,0.911)	0.018^**^	0.730 (0.516,1.033)	0.075^*^	0.826 (0.575,1.187)	0.302
**Outpatient working hours per week (ref: ≤8)**						
~16	0.575 (0.332,0.998)	0.049^**^	0.623 (0.397,0.976)	0.039^**^	0.700 (0.434,1.130)	0.144
~24	0.416 (0.237,0.730)	0.002^***^	0.468 (0.298,0.734)	0.001^***^	0.723 (0.450,1.162)	0.180
~40	0.473 (0.245,0.911)	0.025^**^	0.521 (0.308,0.882)	0.015^**^	0.848 (0.488,1.474)	0.559
>40	0.170 (0.0769,0.375)	<0.001^***^	0.370 (0.215,0.638)	<0.001^***^	0.610 (0.346,1.074)	0.086^*^

*Base outcome: very high PCW physician group*;

* *p < 0.1*;

** *p < 0.05*;

*** *p < 0.01*;

*RRR, relative risk ratio; CI, confidence interval; ref, reference group; N/A, not applicable*.

Compared to males, female physicians were less likely to be assigned into the low [Relative Risk Ratio (RRR) = 0.370, *p* < 0.001], medium (RRR = 0.427, *p* < 0.001) or high (RRR = 0.509, *p* < 0.001) PCW physician groups as compared with the odds of very high PCW physician group. Physicians with higher age were more likely to be assigned into the very high PCW physician group; compared to those aged 20–30 years, physicians with an increased age were less likely to be assigned into the high PCW physician group (RRR = 0.506, *p* = 0.035; RRR = 0.392, *p* = 0.020; RRR = 0.323, *p* = 0.057 < 0.10, respectively) as compared with the odds of very high PCW physician group. Compared to those with a master degree, physicians with undergraduate or junior college degrees were less likely to be assigned into the medium PCW physician group (RRR = 0.653, *p* = 0.030; RRR = 0.456, *p* = 0.089 < 0.10, respectively), and physicians with undergraduate degrees were also less likely to be assigned into the high PCW physician group (RRR = 0.690, *p* = 0.071 < 0.10) as compared with the odds of very high PCW physician group. Compared to those with junior titles, physicians with associate senior titles were more likely to be assigned into the low (RRR = 2.119, *p* = 0.079 < 0.10), medium (RRR = 1.864, *p* = 0.072 < 0.10) or high (RRR = 3.903, *p* < 0.001) PCW physician groups, and physicians with senior titles were also more likely to be assigned into the high PCW physician group (RRR = 2.564, *p* = 0.043) as compared with the odds of very high PCW physician group. Moreover, physicians who worked in the current medical institution for 11–15 or 16–20 years were less likely than those with 6–10 working years in the current medical institution to be assigned into the medium (RRR = 0.627, *p* = 0.063 < 0.10; RRR = 0.526, *p* = 0.015, respectively) or high (RRR = 0.388, *p* = 0.003; RRR = 0.367, *p* = 0.003) PCW physician groups as compared with the odds of very high PCW physician group.

Compared to those from Western China, physicians from Eastern or Central China were more likely to be assigned into medium (RRR = 1.573, *p* = 0.028; RRR = 1.677, *p* = 0.016, respectively) or high (RRR = 1.627, *p* = 0.017; RRR = 1.457, *p* = 0.081 <0.10) PCW physician groups, and physicians from Central China were also more likely to be assigned into the low PCW physician group (RRR = 1.957, *p* = 0.010) as compared with the odds of very high PCW physician group. Physicians who worked in tertiary B hospitals were more likely than those from tertiary A hospitals to be assigned into low (RRR = 2.075, *p* = 0.074 <0.10) or high (RRR = 1.987, *p* = 0.057 <0.10) PCW physician groups. Compared to those worked in Pediatrics, physicians who worked in Internal or Surgical were less likely to be assigned into the low (RRR = 0.451, *p* = 0.062 <0.10; RRR = 0.388, *p* = 0.036, respectively), medium (RRR = 0.490, *p* = 0.046; RRR = 0.433, *p* = 0.027, respectively) or high (RRR = 0.482, *p* = 0.046; RRR = 0.227 *p* < 0.001, respectively) PCW physician groups as compared with the odds of very high PCW physician group.

Moreover, compared to those who worked 41–60 h per week, physicians who worked >60 h per week were less likely to be assigned into the low (RRR = 0.585, *p* = 0.018) or medium (RRR = 0.730, *p* = 0.075 <0.10) PCW physician groups, and physicians who worked no more than 40 h per week were less likely to be assigned into the high (RRR = 0.439, *p* = 0.007) PCW physician group. In addition, physicians with longer outpatient working hours per week were more likely to be assigned into the very high PCW physician group; compared to those with no more than 8 working hours in outpatient practice, physicians with longer outpatient working hours (>8 working hours per week) were less likely to be assigned into the low (RRR = 0.575, *p* = 0.049; RRR = 0.416, *p* = 0.002; RRR = 0.473, *p* = 0.025; RRR = 0.170, *p* < 0.001, respectively) or medium (RRR = 0.623, *p* = 0.039; RRR = 0.468, *p* = 0.001; RRR = 0.521, *p* = 0.015; RRR = 0.370, *p* < 0.001, respectively) PCW physician groups as compared with the odds of very high PCW physician group.

## Discussions

### Principal Findings

Overall, the mean score of PCW tethered to outpatient practice Chinese physicians experienced was 811.30 (SD = 494.98) with concentrating on between 200 and 1,200, together accounting for 80.1% of total samples, according to our developed integrated evaluation model. Multiple linear regression analysis showed that physicians who were female, and from Eastern or Western China, and those who worked more than 60 h per week and longer outpatient hours per week were more likely to experience a higher PCW in outpatient practice.

About 11.2% of Chinese physicians were identified as very high PCW physicians, compared with 11.6% as low PCW physicians, 45.5% medium PCW as physicians and 30.7% as high PCW physicians. This is a result of the combined effect of the mean and standard deviation of the PCW. Multinomial logistic regression analysis further indicated that physicians who were female, older, from Western China, those who had lower educational levels, lower professional titles, and longer working years in the current medical institution, and those who worked in tertiary A hospitals, and Internal or Surgical, and worked >60 h per week and longer outpatient hours per week were more likely to be very high PCW physicians.

### Comparison to Prior Studies

#### Levels of Physician Comprehensive Workload Tethered to Outpatient Practice

This is the first study, to our knowledge, to develop an integrated evaluation model for comprehensively assessing physician workload tethered to outpatient practice based on the combination of objective workload and task-level mental workload also with the consideration of quality of provided medical services and served patient complexity. Previous research often simply used either objective workload or mental workload as a measure of physician workload in various healthcare settings (16, 18, 23, 25, 46), not to mention the comprehensive workload, which might have failed to reflect their real workload. This study revealed that the mean score of PCW Chinese physicians experienced in outpatient practice was 811.30 (SD = 494.98) with concentrating on between 200 and 1,200, together accounting for 80.1% of total samples, according to the integrated evaluation model, which indicates a high level of comprehensive workload. As noted in the Introduction, increased physician workload could adversely affect physicians themselves, and their patients and organizations. Therefore, hospital managers should consider paying more attention to work burden for Chinese physicians in outpatient practice to mitigate the potential negative effects caused by increased workload and even overwhelming workload.

Our analysis results further showed that gender, working hours per week, and outpatient working hours per week were significant factors that influenced PCW in outpatient practice. Similar conclusions were reported in our previous research on physician mental workload that physicians who were female and those who worked more hours per week, with more than 40 outpatient working hours per week were more likely to have high levels of mental workload in outpatient clinics (46). Our analysis also indicated that area was a significant factor that compared to those from Central China, physicians from Eastern or Western China were more likely to experience a higher PCW, whereas it was not significant factor, and other factors such as age, educational level, average monthly income, working years in the current medical institution, hospital level, and self-rated health status all significantly influenced physician mental workload reported in our previous research (46). The effect of area on PCW tethered to outpatient practice is closely related to the current dilemma in medical fields in China. These findings suggest that although there great individual variations existed when mental workload was used as a measure of physician workload, these individual differences among physicians were gradually eliminated when considering these factors as adjustment coefficients for physician workload (including objective workload, quality of provided medical services and served patient complexity), which indicates that on the one hand, mental workload simply used as the measure of physician workload might have failed to reflect the real work burden for physicians, thereby leading to inaccurate identification of individuals with high workload, misleading the targeted interventions of hospital managers, and ultimately resulting in a waste of human resources, and on the other hand, our proposed integrated evaluation model for physician workload might help comprehensively and reliably assess physician workload tethered to outpatient practice, possibly resulting in an enhanced human resources management for hospital mangers.

#### Determinants of Relative Levels of Physician Comprehensive Workload

Internationally, there is still lack of consensus on what should be considered as a threshold value for a high or excessive workload among physicians (35, 41–44), and therefore, how to discriminate and identify individuals with high workload within a specific group is an important topic that hospital managers pay great attention to, especially in current conditions where physicians in their institutions are suffering from adverse effects of increased workload and even overloads. Previous research usually identified individuals with high workload among evaluated physicians using the quartiles (35, 41), threshold values for workload (e.g., 50% of overall workload) (42) or high workload corresponding to composite NASA-TLX scores of >55 (43) or >60 (44) through subjective evaluations, not to mention the comprehensive workload assessments. Even some research on a national survey assessed Chinese physicians' overall workload simply based on the question on a 5-point Likert scale that “I have a heavy workload,” where these physicians who answered “strongly agree” or “agree” were classified as individuals with heavy workload (14). This study, to our best knowledge, is an early study discriminating and identifying individuals with high comprehensive workload among evaluated physicians based on combined effects of the mean and standard deviation of the PCW.

Our analysis showed that four distinctive groups of PCW physicians were identified, and about 11.2% of Chinese physicians were identified as very high PCW physicians, compared with 11.6% as low PCW physicians, 45.5% as medium PCW physicians and 30.7% as high PCW physicians, whereas a much higher share of physicians with heavy workload (64.51%) was reported in a recent national survey from 136 public tertiary hospitals across all 31 provinces of China (14), and a lower share of physicians with high mental workload (33.8%) was reported in our previous research through latent profile analysis (46). This large difference could be explained by the scope of assessments of physician workload related to work tasks, the measurement tool of physician workload, or the determination method for high workload or heavy workload.

Our study further revealed that great individual variations across the four distinctive groups of PCW physicians existed. Despite a shortage of studies comparing individual differences across PCW physicians, several existing studies have pointed to great variations in subtypes of mental workload across individual characteristics among medical workers, thereby resulting in an accurate identification of the characteristics of individuals with high mental workload (46, 47). Our study indicated that gender, age, area, educational level, professional title, working years in the current medical institution, hospital level, department, working hours per week, and outpatient working hours per week were significant predictors of four distinctive groups of PCW physicians, and physicians who were female, older, from Western China, those who had lower educational levels, lower professional titles and longer working years in the current medical institution, and those who worked in tertiary A hospitals, and Internal or Surgical, and worked >60 h per week and longer outpatient hours per week were more likely to be very high PCW physicians in outpatient practice, which is mostly supported by the findings from our previous study on mental workload based on latent profile analyses that these physician characteristics, such as female, younger, lower educational levels, tertiary A hospitals, more working hours per week, more than 40 outpatient working hours per week, and 16–20 working years in the current medical institution were all significantly associated with higher mental workload of physicians while performing physician-patient communication work tasks in outpatient clinics (46). These existing differences across the characteristics suggest that physicians with high mental workload may not necessarily experience a heavy comprehensive workload in outpatient practice, and therefore, for hospital managers, mental workload simply used as the measure of physician workload may not be able to reflect the real workload, and further accurately determine and identify physicians with high workload as individuals who need interventions in priority, ultimately leading to a waste of limited human resources. These findings also suggest that our proposed integrated evaluation model for physician workload could be reliable for comprehensively assessing PCW tethered to outpatient practice to some extent.

With the rapidly aging population with chronic and age-related diseases and its subsequent demands for health care services (1), along with lack of a proportional growth in the number of physicians, increasingly heavy outpatient workload for physicians especially from high-level hospitals (tertiary A hospitals) is still a very prominent issue for Chinese health care system (11). A recent study indicated that from 1998 to 2016, there has been a trend of dramatically increased workload for Chinese physicians, potentially threatening not only their health but also the quality of medical services they provided (48). As noted in the Introduction, increased workload for physicians could have adverse effects on physicians themselves, and their patients and organizations. To reduce these adverse effects caused by increased workload and even overwhelming workload, it's critical to strengthen the assessment and management of physician workload. Given that there is an increased demand for more professional health workers in China (49), accurate identification of physicians with high workload or heavy workload is essential to optimize the allocation of limited human resources. Such a strategy should be based on comprehensively assessing physician workload rather than simply assessing either mental workload or objective workload, or both objective workload and mental workload together. Therefore, an integrated evaluation model for comprehensively assessing physician workload tethered to outpatient practice was developed in this study based on the combination of objective workload and task-level mental workload also with the consideration of quality of provided medical services and served patient complexity. To minimize these adverse effects caused by increased workload, especially in high time-pressure outpatient settings, we suggest that hospital managers should consider setting up a special department to be responsible for monitoring and management of physician comprehensive workload to, in turn, dynamically identify individuals who need interventions in priority. Such an outcome can further help hospital managers to strengthen the dynamical management of human resources in their institutions to, in turn, achieve higher organization performance, while mitigating the potential negative effects caused by increased workload.

## Limitations

There were several limitations mentioned in this study. First, patient satisfaction is an important indicator of measuring the quality of medical services (39), and accordingly was used as a single indicator for measuring the quality of care provided medical services in our developed integrated evaluation model, but its self-reported measurement method by physicians might have resulted in biased measurement errors, and therefore, further research is still needed to improve the measurement accuracy and precision of the quality of care provided medical services in the integrated evaluation model. Second, patient difficulty in the diagnosis and treatment could be simply measured based on the question that “did you have any patients that you perceived as difficult?” reported in previous research (23), and accordingly, the complexity of the patient served by the physician in the diagnosis and treatment was reflected by the ratio of the number of outpatients serviced per day admitted to the hospital by a physician for further diagnosis or treatment to the number of outpatients seen per day by the physician in this study; and if a physician did not service an outpatient who needs to be hospitalized, the ratio would be zero, resulting in zero PCW, which may have limited the generalizability of the ratio as a measure of patient complexity, and therefore, we normalized this ratio and added 1 (that is, 1 represents the reference value) to avoid that the ratio was zero, and our further research is needed to improve the measurement accuracy of seen patient complexity, and thereby further improve the applicability of our proposed integrated evaluation model for physician workload tethered to outpatient practice; and moreover, patient complexity was reflected only by a single indicator, which may have resulted in a measurement error to some extent. Third, quality of provided medical services and served patient complexity were all used as adjustment coefficients included in our proposed integrated evaluation model, and there are still many important factors that influenced physician workload, such as, interruptions and assistants, and therefore, more research is still needed to consider more factors included as adjustment coefficients to improve the reliability and stability of the proposed integrated evaluation model for physician workload. Moreover, some limitations on this cross-sectional national survey have been reported in our previous research (46), for example, data collection was self-reported by participating physicians via the online survey, and accordingly, there was no guarantee that the participating physicians in this study filled out the survey questionnaire just after finishing the provision of the outpatient services in outpatient practice, which might have resulted in a recall bias; and some lower responsiveness was received in some selected hospitals, which may have limited the sample size to some extent, and therefore, to improve the scale of the targeted physicians, we invited the outpatient managers in each selected hospital to play the role of the project manager in their hospitals during this online survey, and encouraged all participants who completed the questionnaire to share the survey website link to their Wechat Circle of Friends, WeChat or Tencent QQ group, where some physicians who met the inclusion criteria for the targeted population could be invited and participate in this questionnaire survey.

## Conclusions

Chinese physicians experienced high levels of comprehensive workload in outpatient practice; and those who were female, and from Eastern or Western China, and those worked > 60 h per week and longer outpatient hours per week were more likely to experience a higher PCW. About 11.2% of Chinese physicians were identified as very high PCW physicians, compared with 11.6% as low PCW physicians, 45.5% as medium PCW physicians and 30.7% as high PCW physicians. Great individual variations in four distinctive groups of PCW physicians existed. Physicians who were female, older, from Western China, those who had lower educational levels, lower professional titles and longer working years in the current medical institution, and those who worked in tertiary A hospitals, and Internal or Surgical, and worked >60 h per week and longer outpatient hours per week were more likely to be very high PCW physicians. Our work has a potential application for comprehensively assessing physician workload tethered to outpatient practice and could provide a solid foundation for hospital managers to further determine and accurately identify physicians with high workload, who would otherwise be missed in either objective workload or mental workload.

## Data Availability Statement

The datasets used and/or analyzed during the current study are available from the corresponding author on a reasonable request. Requests to access the datasets should be directed to hyh288@hotmail.com.

## Ethics Statement

Ethics approval was obtained from the Ethics Committee of Tongji Medical College of Huazhong University of Science and Technology (No. IORG0003571). All the survey data were kept confidential and anonymous.

## Author Contributions

YH designed the study, obtained funding, participated in the collection, and performed revisions of the manuscript. DL contributed to the design of this study, the acquisition, analysis and interpretation of survey data, and drafted the manuscript. SL and CL participated in the data cleaning, contributed to the interpretation of the results, and performed revisions of the manuscript. YZ and JZ contributed to the interpretation of the results and performed revisions of the manuscript. JL and ZZ were involved in data cleaning and contributed to the interpretation of the results. All authors have read and approved the final version of the manuscript.

## Funding

This study was supported by the National Natural Science Foundation of China (grant number 71774062).

## Conflict of Interest

The authors declare that the research was conducted in the absence of any commercial or financial relationships that could be construed as a potential conflict of interest.

## Publisher's Note

All claims expressed in this article are solely those of the authors and do not necessarily represent those of their affiliated organizations, or those of the publisher, the editors and the reviewers. Any product that may be evaluated in this article, or claim that may be made by its manufacturer, is not guaranteed or endorsed by the publisher.
